# Supplementary material for: Biobased and biodegradable films exhibiting circularly polarized room temperature phosphorescence
Source: Nat Commun. 2024 Mar 15;15:2375. doi: 10.1038/s41467-024-45844-5 (PMC10943238; doi:10.1038/s41467-024-45844-5)
Supplement: Supplementary file 1 — Supplementary Information [file 41467_2024_45844_MOESM1_ESM.pdf]

Supplementary Information

Biobased and biodegradable films exhibiting circularly polarized room temperature phosphorescence

Mengnan Cao[1], Yiran Ren[2], Yue Wu[*,2], Jingjie Shen[1], Shujun Li[*,1], Zhen-Qiang Yu[2], Shouxin Liu[1], Jian Li[1], Orlando J. Rojas[*,3,4,5e] and Zhijun Chen[*,1]

[1] Key Laboratory of Bio-based Material Science and Technology of Ministry of Education, Northeast Forestry University, Harbin 150040, China. E-mail: chenzhijun@nefu.edu.cn; lishujun@nefu.edu.cn

[2] College of Chemistry and Environmental Engineering, Shenzhen University, Shenzhen 518071, China. E-mail: wuyue@szu.edu.cn

[3] Bioproducts Institute, Department of Chemical & Biological Engineering, University of British Columbia, Vancouver, British Columbia, Vancouver, BC V6T 1Z3, Canada. E-mail: orlando.rojas@ubc.ca

[4] Department of Chemistry, University of British Columbia, Vancouver, BC V6T 1Z1, Canada.

[5] Department of Wood Science, University of British Columbia, Vancouver, BC V6T 1Z4, Canada.

Supplementary Figures

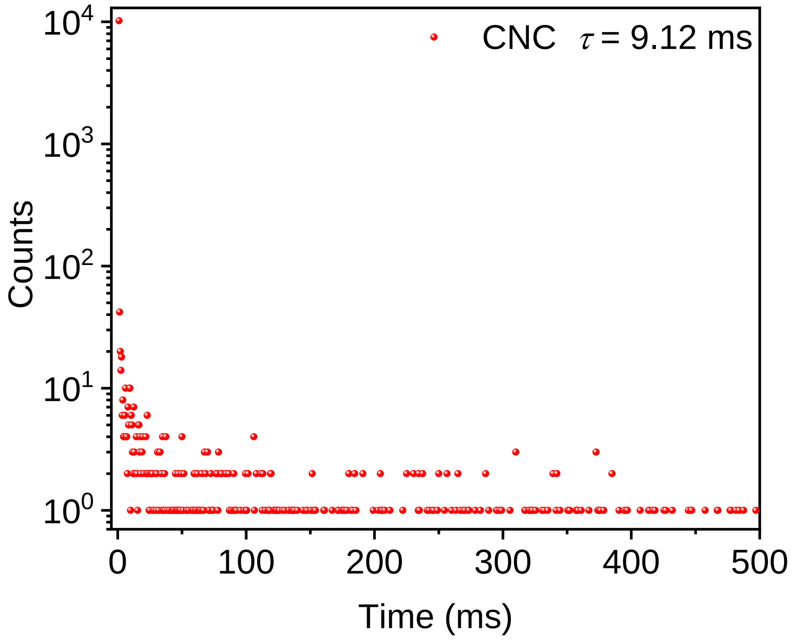

Supplementary Figure 1: Lifetime of CNC emission at 520 nm at 298 K.

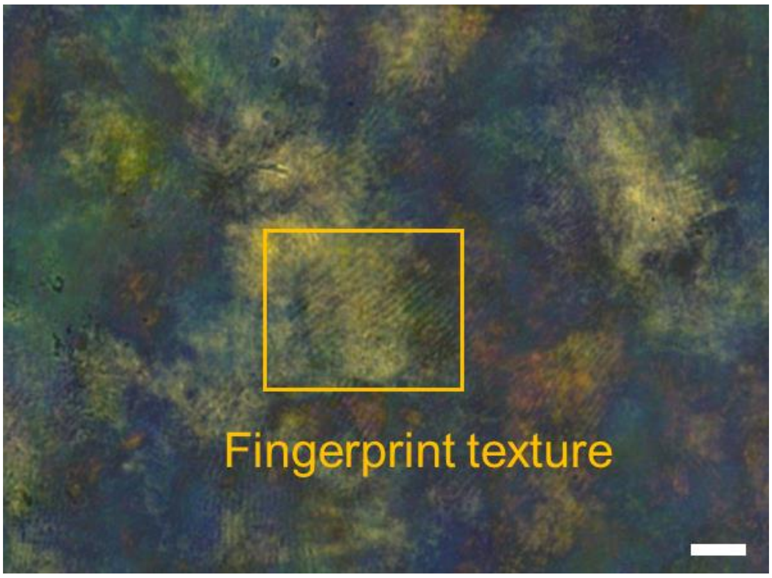

Supplementary Figure 2: Polarizing optical microscope (POM) image of CNC film showing fingerprint, scale bar =200 μm.

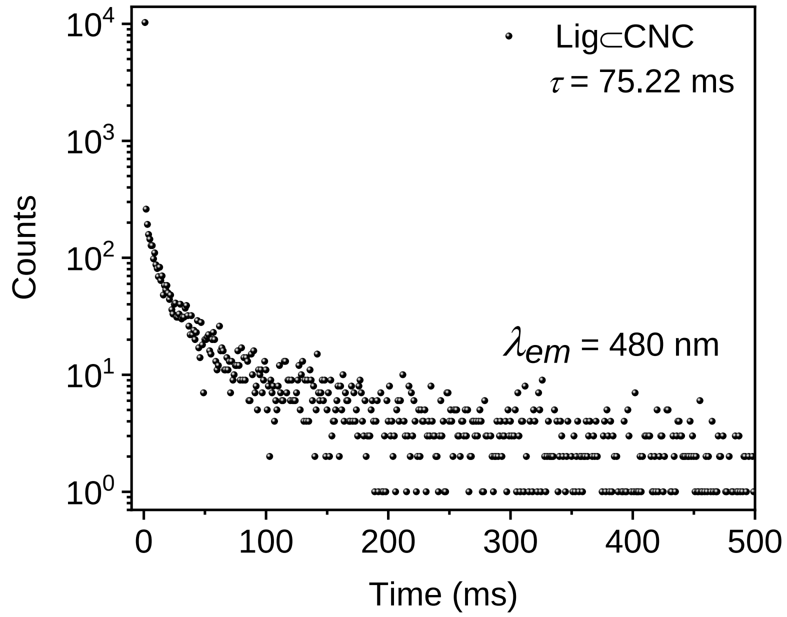

Supplementary Figure 3: Luminescence lifetime of Lig⊂CNC (λ_{em} = 480 nm, 298 K).

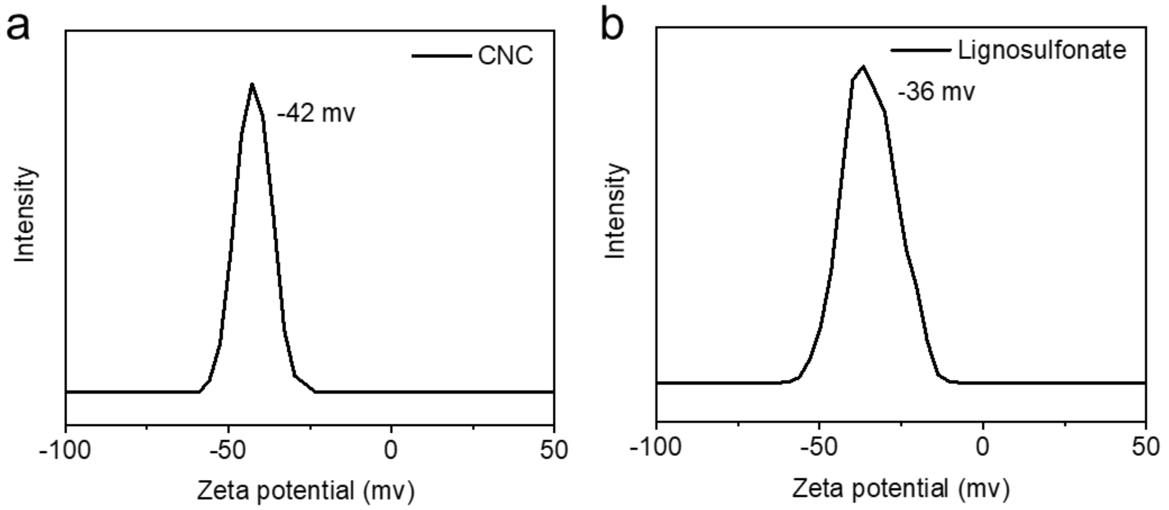

Supplementary Figure 4: Zeta potential of CNC and lignosulfonate.

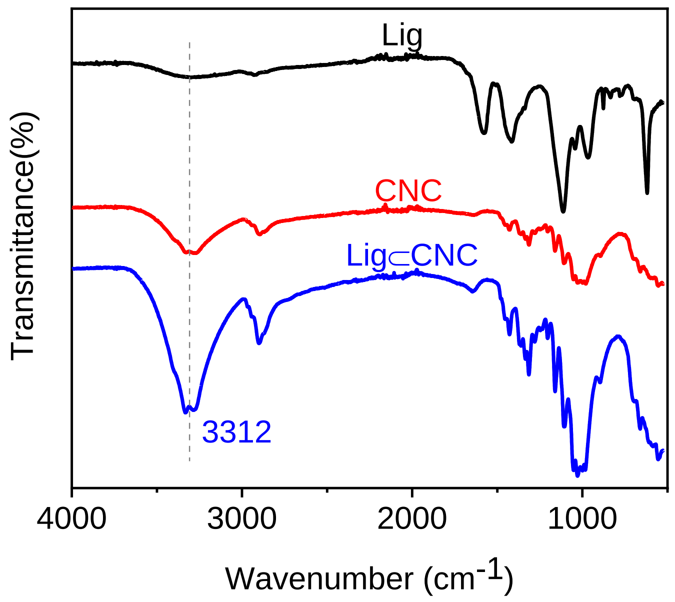

Supplementary Figure 5: FTIR spectra of lignosulfonate, CNC and Lig⊂CNC.

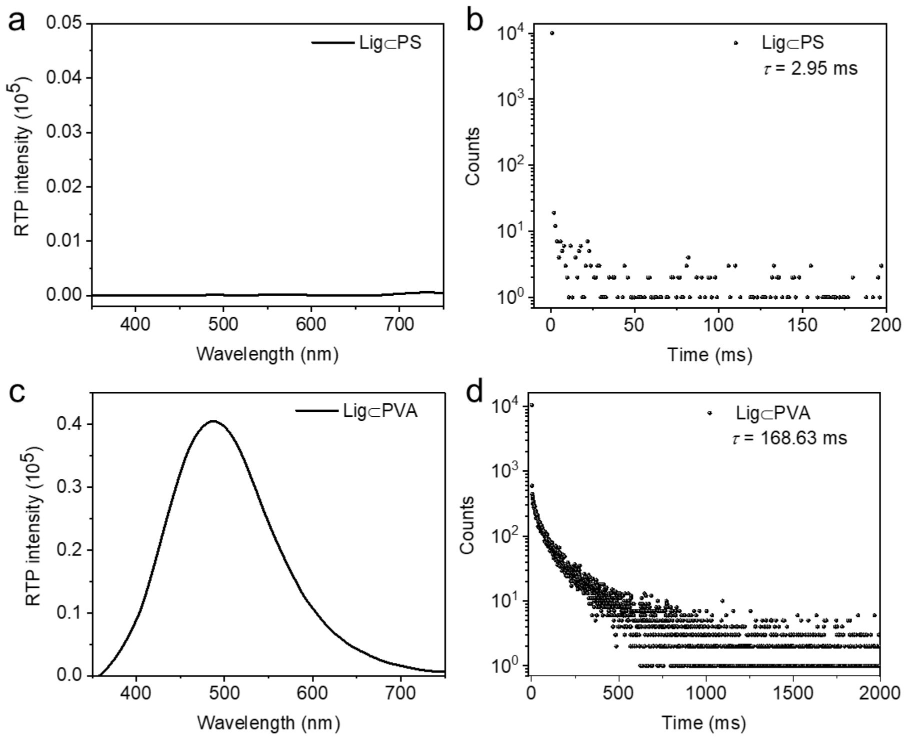

Supplementary Figure 6: Phosphorescence spectra and lifetime of Lig⊂PS and Lig⊂PVA films. (a) Phosphorescence spectra and (b) Lifetime of a Lig ⊂PS film. (c) Phosphorescence spectra and (d) lifetime of a Lig ⊂PVA film (PS: polystyrene and PVA: polyvinyl alcohol).

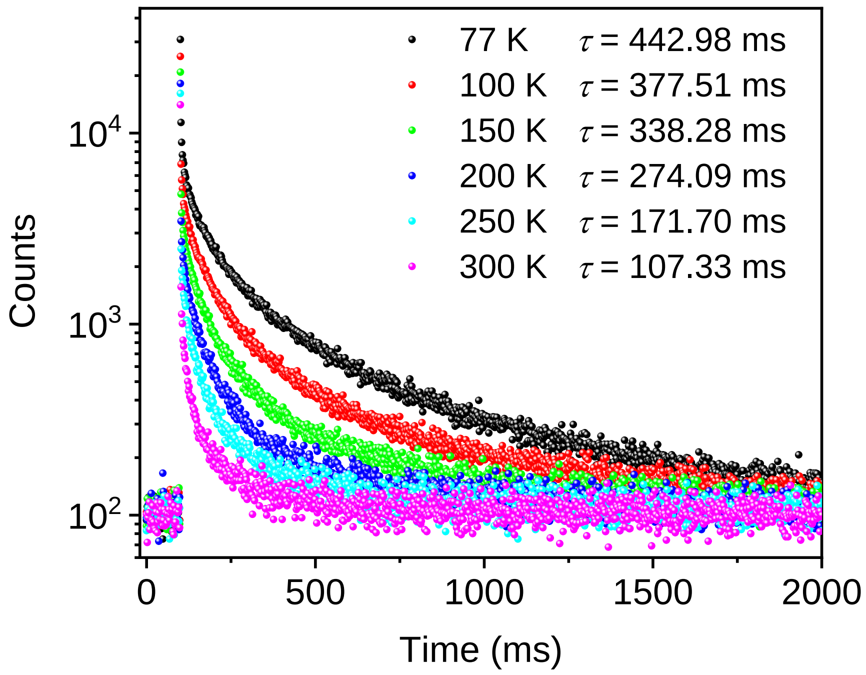

Supplementary Figure 7: Temperature-dependent luminescence lifetime of Lig⊂CNC at 520 nm.

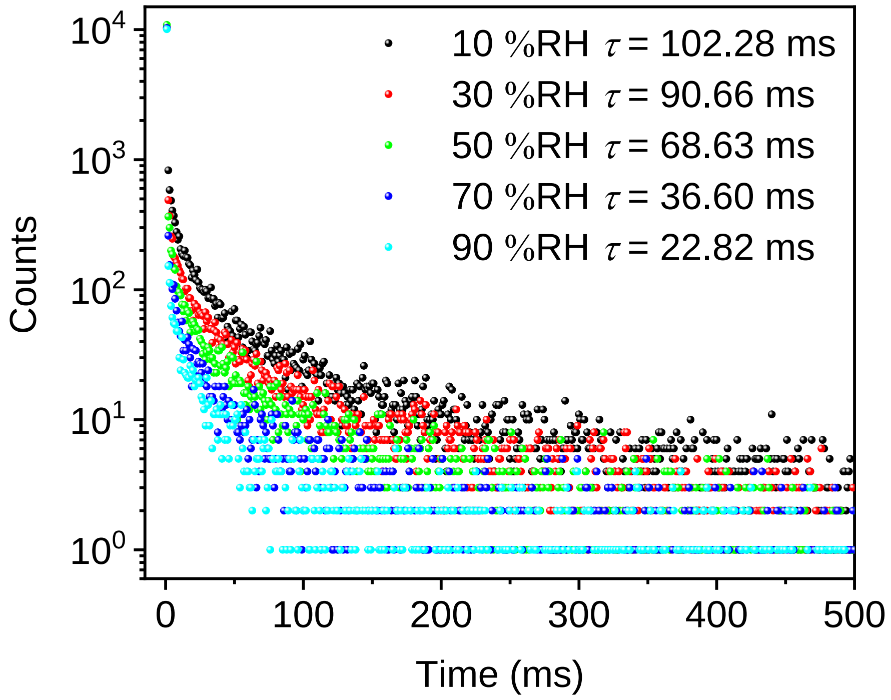

Supplementary Figure 8: Luminescence lifetime of Lig⊂CNC at 520 nm under different humidity environments.

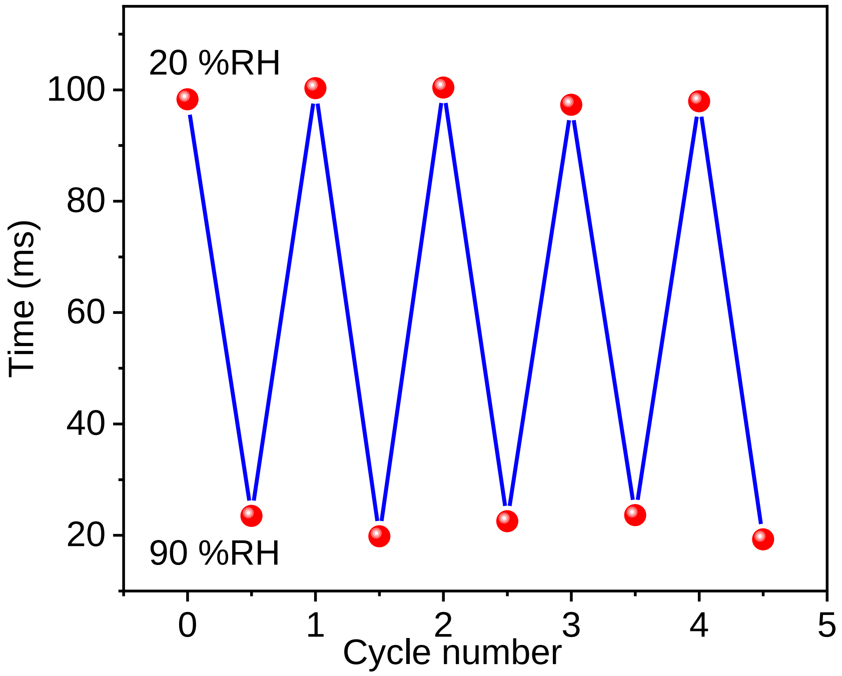

Supplementary Figure 9: Lifetime of Lig⊂CNC under cyclic conditioning (humid (90 %RH) and dry (20 %RH) environments).

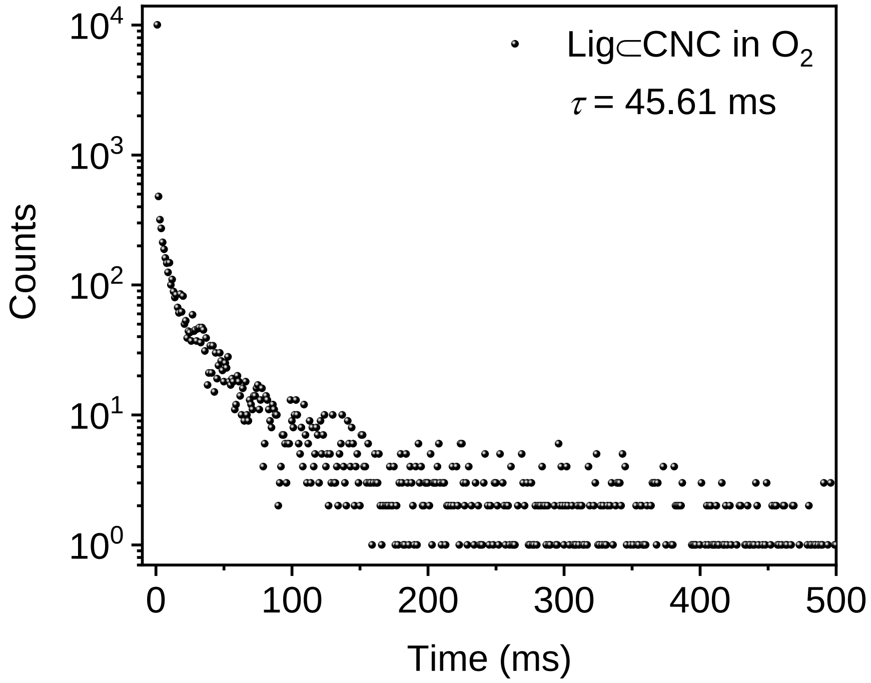

Supplementary Figure 10: Luminescence lifetime of Lig⊂CNC at 520 nm in an environment with 90% O_2.

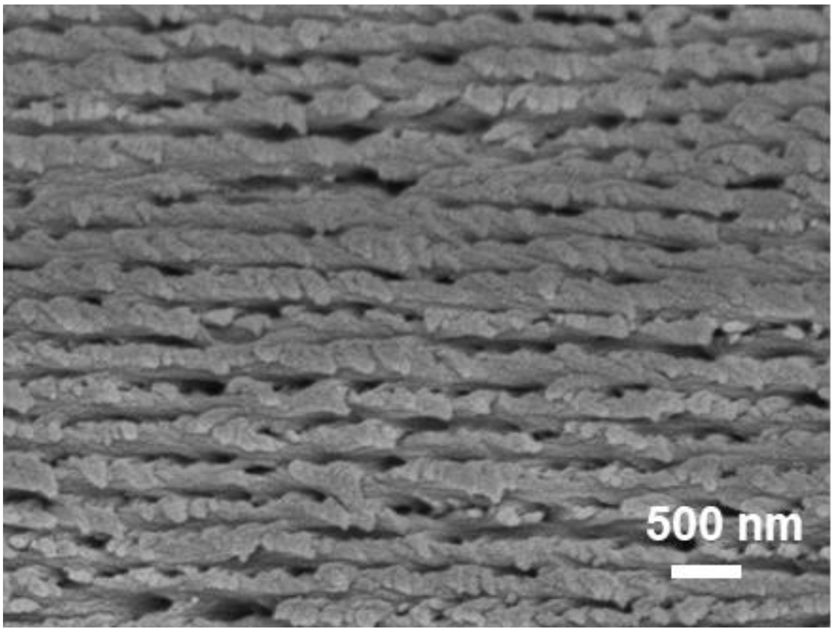

Supplementary Figure 11: Scanning electron microscopy (SEM) cross-section image of a CNC film.

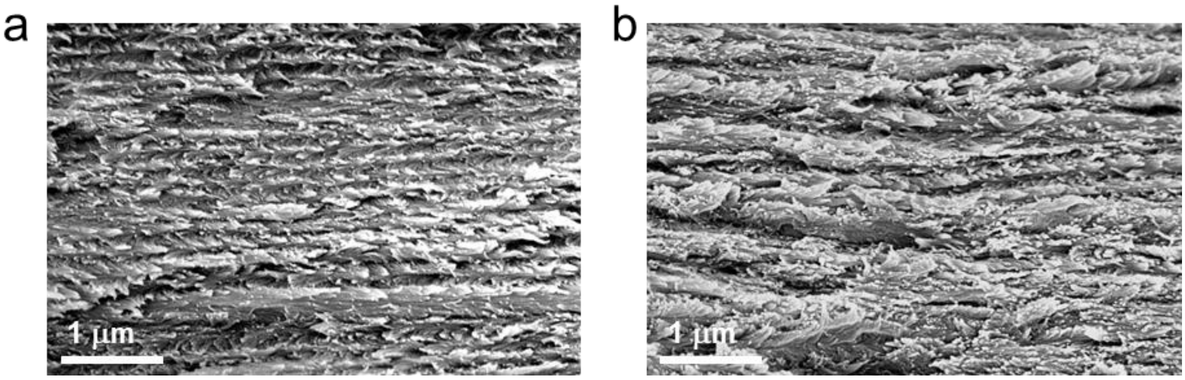

Supplementary Figure 12: Scanning electron microscopy (SEM) cross-section images of (a) Lig⊂CNC film (f_{Lig} =1%) and (b) Lig⊂CNC film (f_{Lig} =2%). Scale bar = 1 μm.

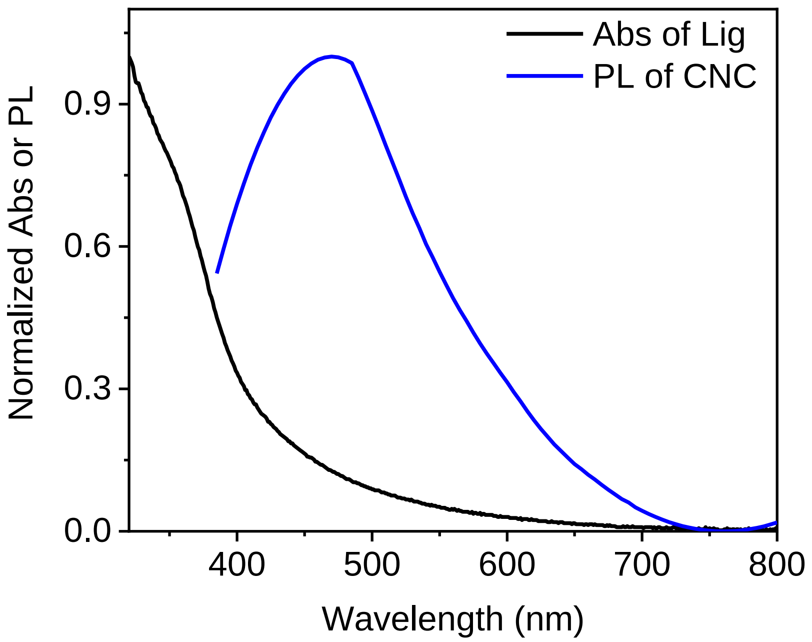

Supplementary Figure 13: UV-vis absorption spectra of lignosulfonate (black profile, left) and luminescence spectra of CNC (blue profile, right).

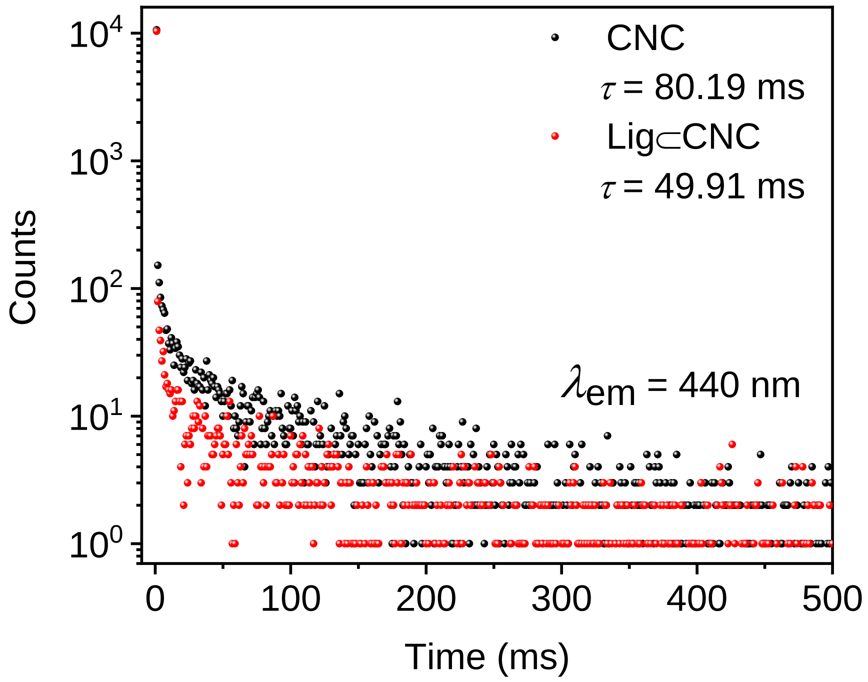

Supplementary Figure 14: Luminescence lifetime of CNC and Lig⊂CNC (λ_{em} = 440 nm) under 310 nm excitation. According to the Förster resonance energy transfer (FRET) efficiency equation ($E = 1 - \tau_{Donor\text{-}Acceptor}/\tau_{Donor}$), the calculated FRET efficiency is 37.8%.

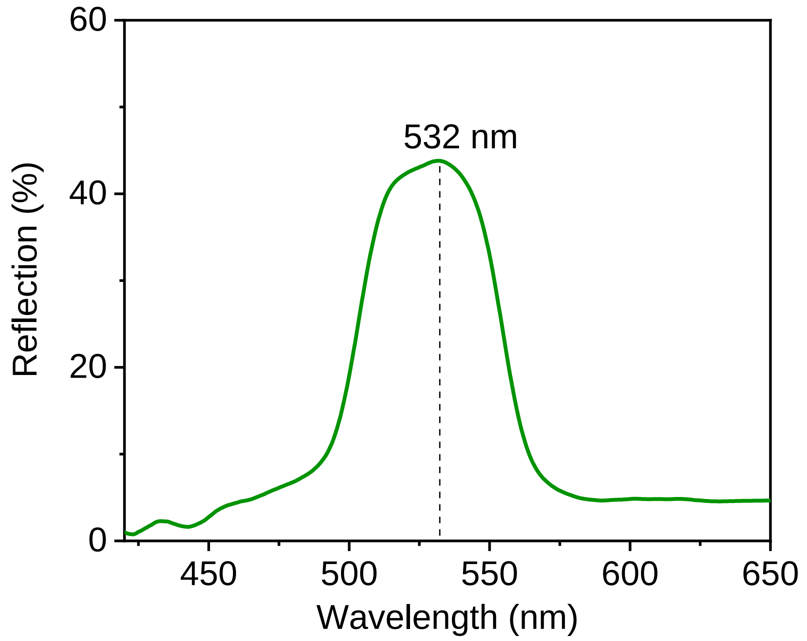

Supplementary Figure 15: Reflection of chiral liquid crystal assembly (2.33 w% R5011 in TEB300) indicating strong reflection, ranging from 500 to 550 nm.

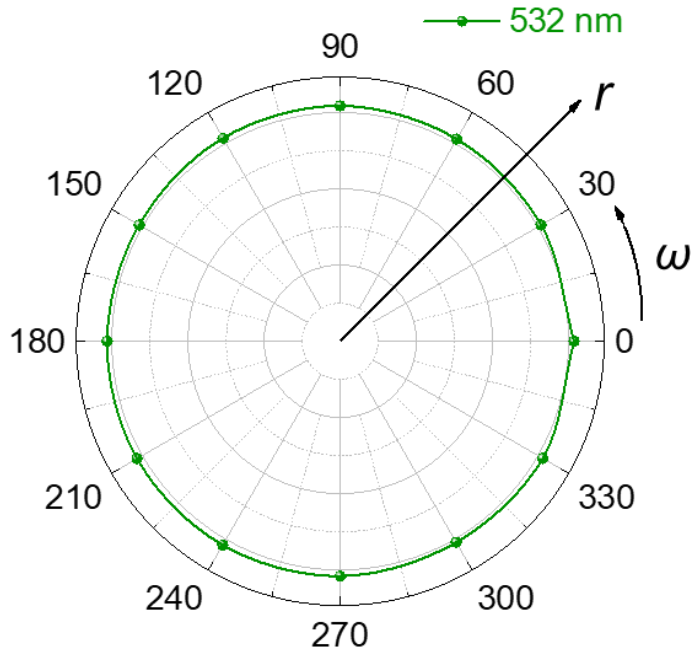

Supplementary Figure 16: Transmission intensity of linearly polarized light at 532 nm across a chiral liquid crystal (LC) system (2.33 w% R5011 in TEB300).

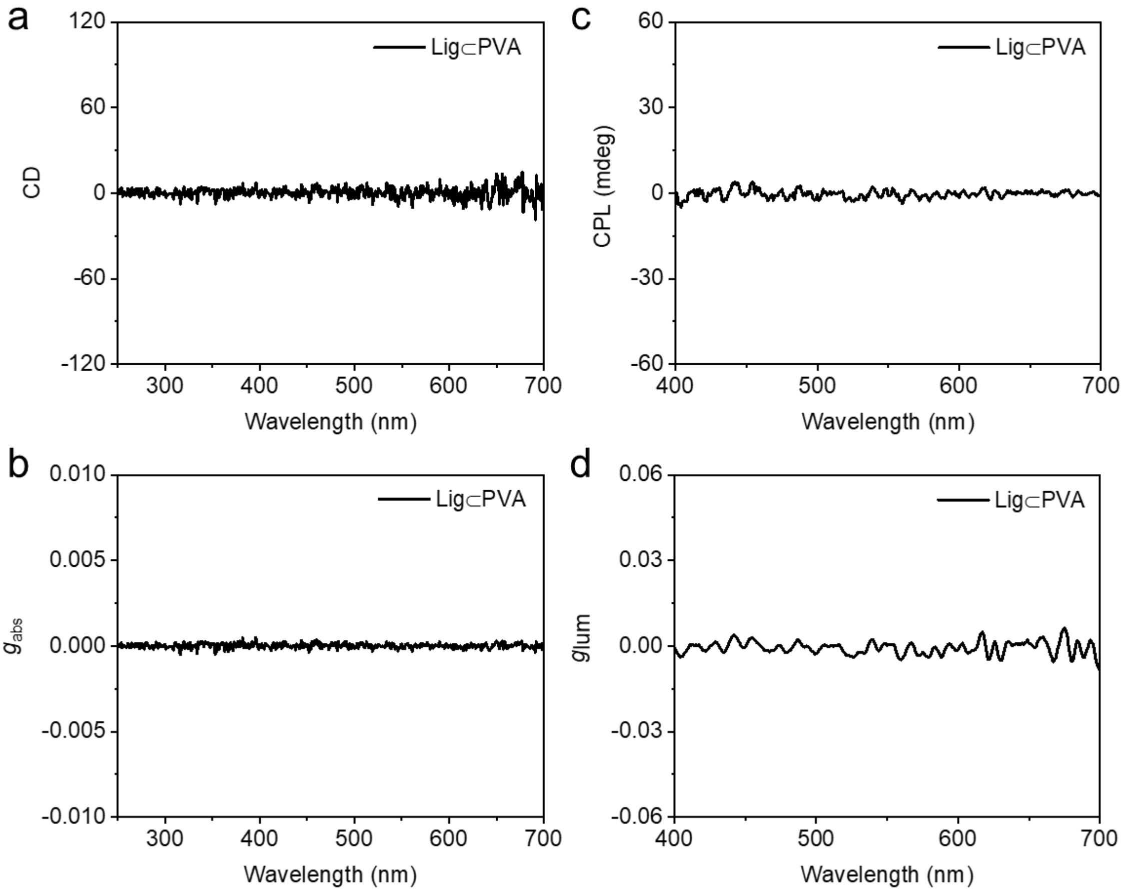

Supplementary Figure 17: Chiroptical properties of Lig⊂PVA films. (a) CD and (b) g_{abs} spectra for Lig⊂PVA thin-film; (c) CPL and (d) g_{lum} spectra for Lig⊂PVA with a 365-nm light excitation.

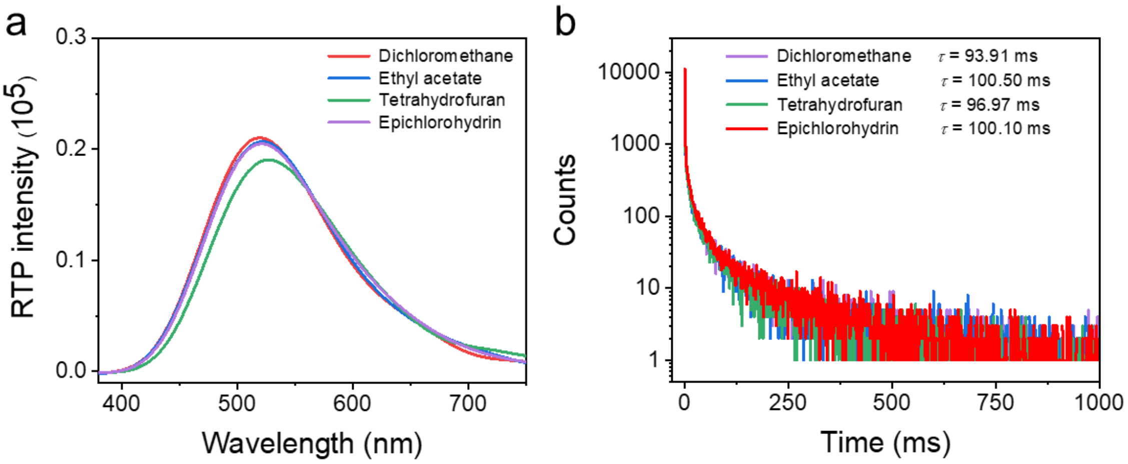

Supplementary Figure 18: RTP intensity and lifetime of Lig⊂CNC in different solvents.

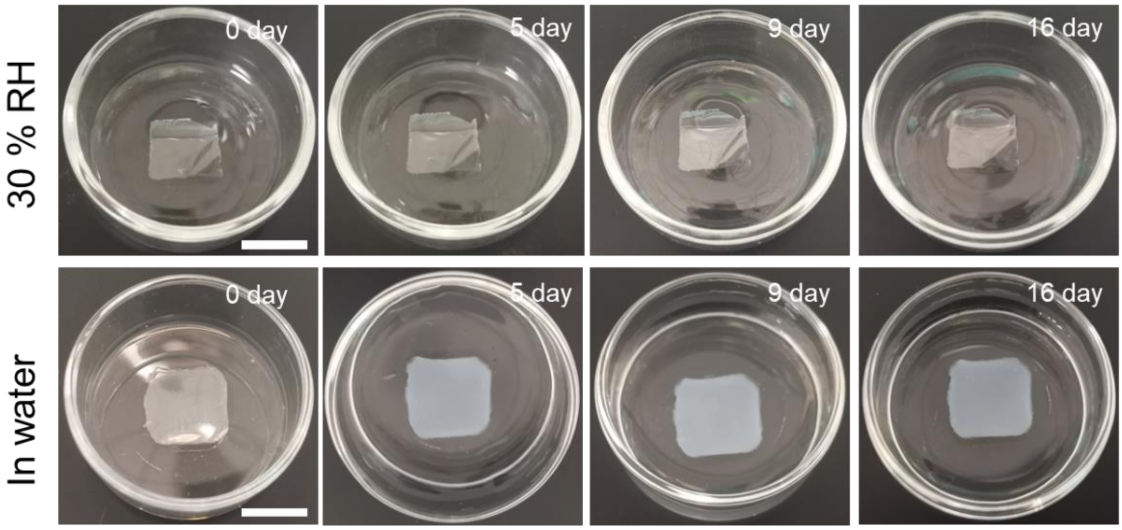

Supplementary Figure 19: Solubility of Lig⊂CNC film in 30 %RH and immersed in water. Scale bar = 1 cm.

Supplementary Tables

Supplementary Table 1: Inputs and outputs in the process of lignosulfonate production.

**Input**		**Unit**	**Amount**
Sawdust	mass	g	51.3425
Branches	mass	g	10.2575
Straw	mass	g	9.7625
Hydrogen peroxide	mass	g	2.0625
Sodium hydroxide	mass	g	0.11
Water	mass	g	4.18
Electricity	energy	kW*h	0.0039875
**Output**			
Lignosulfonate	mass	g	77
Waste			
Wastewater	mass	g	0.51519908
Emissions to air			
Dust	mass	g	0.007719085
Emissions to water			
CODcr	mass	g	0.0000254375
NH_3-N	mass	g	0.00000256

Supplementary Table 2: Inputs and outputs in the Lig⊂CNC process.

Input		Unit	Amount
Filter paper	mass	g	923
Lignosulfonate	mass	g	77
Sulfuric acid	mass	g	2560
Water	mass	g	2400
Electricity	energy	kW*h	0.5
**Output**			
Lig⊂CNC	mass	g	1000

Supplementary Table 3: Inputs and output of C-wood process.

Input		Unit	Amount
wood	mass	g	900
magnesium chloride	mass	g	200
Water	mass	g	1000
Electricity	energy	kW*h	12.8
**Output**			
C-wood	mass	g	1000

Supplementary Table 4: Inputs and output of a-CDs/BA process.

Input		Unit	Amount
Citric Acid	mass	g	143
Boric acid	mass	g	857
Water	mass	g	11700
Electricity	energy	kW*h	29.3
Output			
a-CDs/BA	mass	g	1000

Supplementary Table 5: Inputs and output of $Gd_3Al_2Ga_3O_{12}:Ce^{3+}$ process.

Input		Unit	Amount
Gadolinium oxide	mass	g	581
Aluminum oxide	mass	g	110
Gallium	mass	g	303
Cerium oxide	mass	g	7
Electricity	energy	kW*h	32
**Output**			
$Gd_3Al_2Ga_3O_{12}:Ce^{3+}$	mass	g	1000

Supplementary Table 6: Life cycle assessment results of Lig⊂CNC, C-wood, a-CDs/BA and $Gd_3Al_2Ga_3O_{12}:Ce^{3+}$ process.

Category	Unit	Lig⊂CNC	C-wood	a-CDs/BA	$Gd_3Al_2Ga_3O_{12}:Ce^{3+}$
GWP	kg CO_2-eq	3.625646	11.975257	31.262224	133.36504
